# Design of Multi-Mode Antenna Array for Use in Next-Generation Mobile Handsets

**DOI:** 10.3390/s20092447

**Published:** 2020-04-25

**Authors:** Naser Ojaroudi Parchin, Haleh Jahanbakhsh Basherlou, Raed A. Abd-Alhameed

**Affiliations:** 1 Faculty of Engineering and Informatics, University of Bradford, Bradford BD7 1DP, UK; R.A.A.Abd@bradford.ac.uk; 2 Bradford College, Bradford BD7 1AY, UK; Hale.Jahanbakhsh@gmail.com; 3 Department of Communication and Informatics Engineering, Basra University College of Science and Technology, Basra 61004, Iraq

**Keywords:** 5G, future handsets, modified PIFA, multi-antenna system, multi-band operation

## Abstract

In this study, a new design of a tri-band multiple-input–multiple-output (MIMO) antenna array is proposed for fifth-generation (5G) cellular systems. Its structure is composed of eight identical planar-inverted F antenna (PIFA) elements placed at different edge corners of the handset mainboard with overall dimensions of 150 × 75 mm^2^. The PIFA elements and ground plane of the MIMO antenna system are arranged on the back layer of the platform, which makes the design easy to integrate with the handset circuit. For S_11_ ≤ −10 dB, the radiation elements of the MIMO design operate at the frequency ranges of 2.5–2.7 GHz, 3.4–3.75 GHz, and 5.6–6 GHz covering the long-term evolution (LTE) 41, 42/43, and 47 operation bands, respectively. The array achieves better than 15 dB return loss results across the three operating bands. The presented antenna array not only exhibits multi-band operation but also generates the polarization diversity characteristic, which makes it suitable for multi-mode operation. The proposed antenna array was simulated and experimentally tested. Fundamental characteristics of the proposed design are investigated. It offers three band S-parameters with acceptable isolation and dual-polarized radiation with quite good efficiency and gain results. Besides this, the total active reflection coefficient (TARC) and envelope correlation coefficient (ECC) results of the PIFAs are very low over the bands. In addition, the radiation characteristics of the MIMO antenna in the presence of the user and handset components are studied. Moreover, a new and compact phased array millimeter-wave (MM-Wave) antenna with broad bandwidth and end-fire radiation is introduced which can be easily integrated into the smartphone antenna system. Due to its good performance and simple structures, the proposed smartphone antenna array design is a good candidate for future multi-mode 5G cellular applications.

## 1. Introduction

With the rapid evolution of wireless communications, the 5G network has received a great deal of attention from both academia and industry, with many reported efforts and research outputs [1,2,3]. Significant improvements will be made in different areas, including the data rate speed and resolution, mobility, latency, etc. Multiple-input–multiple-output (MIMO) technology with multiple antennas is a promising technology to obtain the requirements of 5G communications [4,5,6]. To date, 2 × 2 MIMO systems have been successfully employed for 4G mobile networks, and a larger number of antenna elements is expected to be applied for 5G communications [7,8]. The 5G system is predicted to possess an aggregate data rate 1000 times faster than 4G, and it has better link reliability. Thus, compared with the 4G MIMO antenna systems, at least six to eight antenna elements are integrated into a mobile terminal for 5G massive MIMO to provide good diversity and multiplexing gain [9]. This can enhance the channel capacity and link system reliability [10,11]. The greater number of antennas could make it more resistant to intentional jamming and interference. Through spatial diversity and spatial multiplexing, larger channel capacity and better communication reliability can be achieved. Therefore, the multi-antenna system is much more capable of resisting multipath fading and improving data throughput [12]. The 5G network also needs fundamental technologies to enable small cells, beamforming, full duplexing, MIMO, and millimeter-wave (MM-Wave).

For sub-6 GHz 5G cellular communications, LTE band-41 (2.6 GHz), band-42 (3.5 GHz), band-43 (3.7 GHz), and band-47 (5.8 GHz) are the main important candidate frequency bands [13]. Due to the available radio frequency (RF) circuit and test system, 2.6 GHz LTE can be considered as a default for future mobile communications, and it has recently attracted a great deal of interest. Besides this, 3.4–3.8 GHz (LTE band 42/43) is also recognized by many countries as a first step in demonstrating 5G systems. To further support more potential sub-6 GHz frequency bands, LTE band 47, which is also known as the wireless wide area network (WLAN) operation band, can be considered for 5G massive MIMO antenna design [14,15].

Several smartphone antenna designs with MIMO systems have been proposed recently [16,17,18,19,20,21,22,23,24,25,26,27,28,29,30,31,32]. However, all of these designs either cover only a single-band operation frequency or use a few antenna elements with large sizes which could occupy a huge space of the mainboard. The 5G handset antenna designs introduced in [16,17,18,19,20,21,22,23,24] only cover a single frequency band. In [25,26,27,28,29], dual-band or wideband arrays are proposed to support two 5G spectrums. Only a few handset antennas with tri-band function are reported in [30,31,32] for handset applications. However, these antennas have double/quad antenna elements or do not cover important bands such as 2.6 GHz. In this study, we introduced a new MIMO antenna with eight-element planar-inverted F antenna (PIFA) elements which, unlike the reported designs, can cover multi-frequency bands simultaneously. In addition, due to the large number of radiators, the proposed handset antenna can be applied for massive MIMO communication [33]. Furthermore, the proposed handset antenna generates the polarization diversity characteristic, which supports both vertical and horizontal polarizations [34,35,36]. The modified PIFA radiation elements of the design are employed at four corners of the printed circuit board (PCB) to operate at three different frequencies covering the LTE 2600, 42/43, and 47 operation bands. The proposed PIFA array system operates at three different bands—2.6, 3.6, and 5.8 GHz—of sub-6 GHz 5G cellular networks. It exhibits good properties in terms of the fundamental characteristics and could be used in future handsets.

Apart from the sub-6-GHz spectrum, 5G smartphones are also expected to support the MM-Wave spectrum [37]. Compact antennas arranged as an array can be employed in different portions of a smartphone PCB to form linear phased arrays with high gain and directional radiation beams [38,39,40]. In contrast to conventional antennas, such as patch, slot, or monopole antennas, end-fire antennas are more suitable to achieve the required radiation coverage [41,42]. Phased array antennas with high performance are highly desirable for MM-Wave 5G communications as they can increase the radiation and the connectivity of the system [43,44,45]. In addition to the proposed MIMO antenna, a new and compact phased array millimeter-wave (MM-Wave) antenna with broad bandwidth and end-fire radiation is introduced for 28 GHz applications. Its configuration is composed of eight loop dipole resonators with pairs of directors arranged in a linear form which can be easily integrated into the smartphone antenna system. The following sections present the design details, single-element performance, characteristics of the tri-band MIMO antenna, and the 28 GHz phased array.

## 2. Design and Configuration of the Proposed 5G Antenna Array

**Figure 1:**
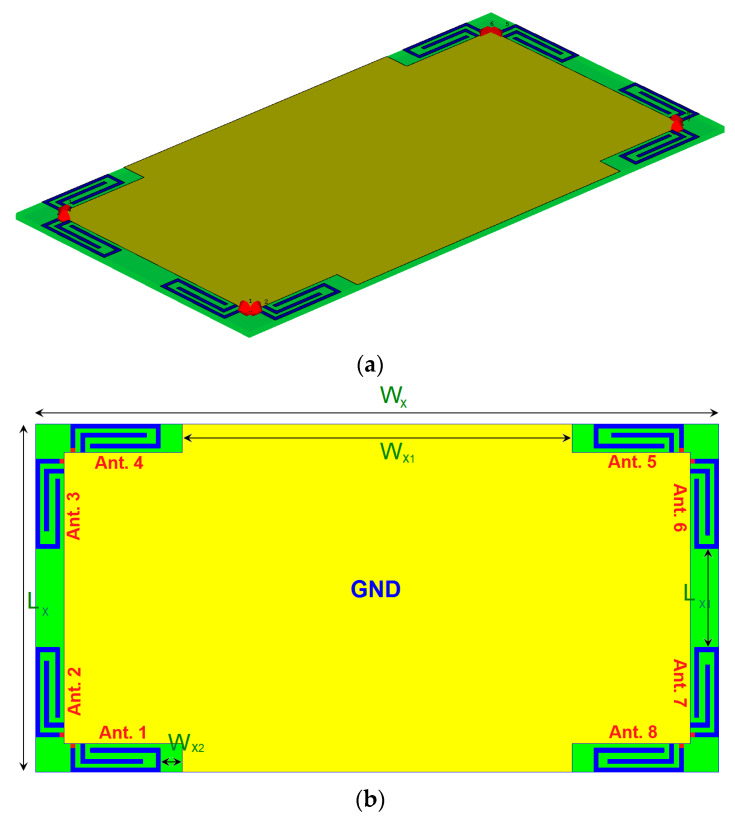
(**a**) Side and (**b**) back views of the multi-mode antenna array.

The schematic of the designed MIMO handset antenna is plotted in Figure 1. As shown, it is composed of four PIFA pairs that have been deployed at different corners of the mainboard. The presented handset antenna is designed on an FR-4 dielectric with a relative permittivity of 4.4, loss tangent of 0.026 and a thickness of h_x_ = 1.6 mm. Each PIFA element is fed by a 50 ohm discrete feeding technique extended from the ground plane to the antenna feedline. The values of the design details are listed in Table 1.

## 3. Characteristics of the Single-Element/Multi-Band PIFA Resonator

**Figure 2:**
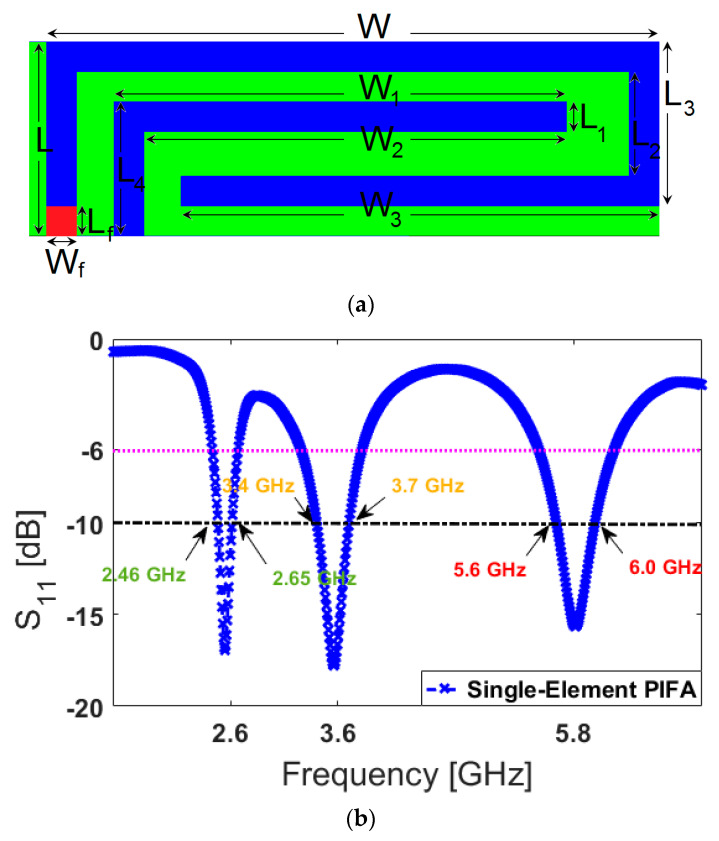
(**a**) Transparent view of the planar-inverted F antenna (PIFA) and (**b**) its S_11_ performance.

The PIFA is a compact size antenna radiator that provides omnidirectional radiation patterns and can be used in hand-held devices [46,47,48]. The conventional PIFAs exhibit single-band operation. However, the modified designs can cover multi-frequency bands for multi-mode operation [49]. The configuration of the PIFA element is depicted in Figure 2a. Its structure is composed of an open-loop resonator with an L-shaped strip protruding from the ground plane. As shown, it has a low profile with the dimension of W × L. A 50 Ω discrete feeding port is employed to excite the antenna. The computer simulation technology (CST) software is used to investigate the properties of the designed mobile-handset antenna [50].

The main motive behind the modified PIFA is to obtain a compact antenna element which can support different frequencies and could be integrated with a mainboard circuit while occupying a small clearance. Figure 2b illustrates the simulated reflection coefficient (S_11_) characteristic of the PIFA element. As shown, the antenna operates at 2.6, 3.6, and 5.8 GHz and provides wide impedance bandwidths at these frequencies.

**Figure 3:**
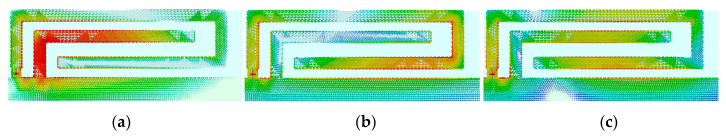
Surface current densities at (**a**) 2.6 GHz, (**b**) 3.6 GHz, and (**c**) 5.8 GHz.

In order to justify the tri-band function of the design, the simulated current densities of the modified PIFA element at different operation frequencies are illustrated in Figure 3. It is worth mentioning that the maximum scaling for all figures is the same. At 2.6 GHz (first resonance), as can be seen, the L-shaped strip has high current densities with the maximum distribution. Additionally, the current flow reverses on the interior edge of the surrounded open loop [51]. It is evident that the second resonance of the antenna S_11_ has been achieved using the open-loop resonator as it appears very active at 3.6 GHz. The third resonance can be considered as the second harmonic of the first resonance [52]. As shown in Figure 3c, the current distribution is almost equal around the L-shaped strip and the open-loop resonator. Nevertheless, some coupling and interactions between the employed parasitic strip of the modified PIFA can be discovered which could affect the frequency response of the antenna [53].

The main motive behind the PIFA design is to obtain a low-profile and multi-mode radiator with the possibility of integration in the mobile-handset mainboard. The S_11_ characteristics of the modified PIFA antenna can be adjusted by changing the values of the fundamental antenna parameters [54,55]. The first resonance (at 2.6 GHz for the low-band) is mainly determined by the L-shaped strip. The second and third resonances (at 3.6 GHz and 5.8 GHz) depends on the main resonator (open-loop). Therefore, the circumference lengths of the resonators can satisfy the dielectric wavelength at the corresponding frequency points [56].

**Figure 4:**
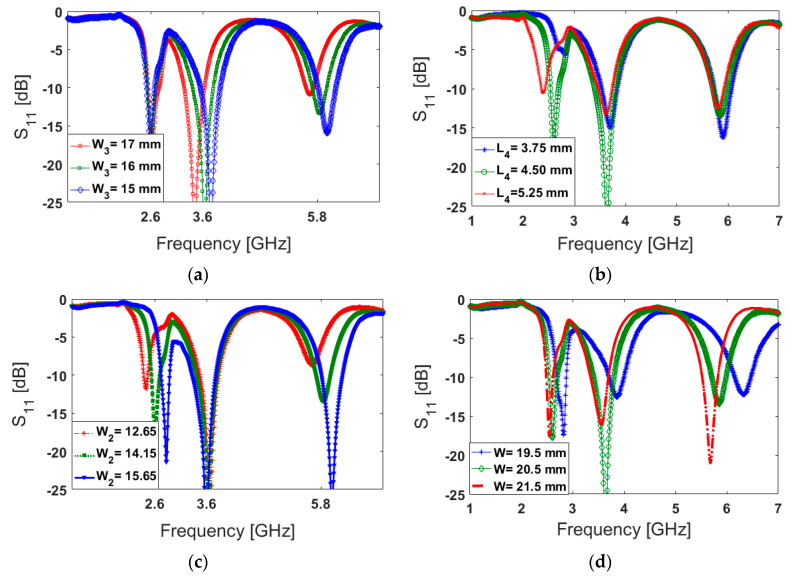
The S_11_ characteristics for different sizes of (**a**) W_3_, (**b**) L_4_, (**c**) W_2_, and (**d**) W.

Figure 4 illustrates the antenna S_11_ characteristic of varying design parameters including W_3_, L_4_, W_2_, and W. In the simulation of the designed antenna, when one parameter changes, the rest of the parameters are kept the same as listed in Table 1. The antenna S_11_ results for different values of W_3_ are illustrated in Figure 4a. As evident from the figure, there is very little impact on the first resonance, while the second and third resonant frequencies are influenced and tuned to higher frequencies. Figure 4b shows that L_4_ (unlike W_3_) has a significant impact on the first resonance frequency and little effect on the second and third resonance frequencies. Figure 4c shows the effects of *W*_2_ (length of L-shaped strip) on the S_11_ of the antenna. It can be observed that as *W*_2_ decreases from 12.65 to 15.65 mm, the first and third resonances at 2.6 GHz and 5.8 GHz shift up to higher frequencies, while very little variation is observed at the middle resonance frequency (3.6 GHz). The antenna S_11_ characteristic of the antenna at different frequencies is also highly dependent on the length of the open-loop resonator (W). As shown in Figure 4d, changing the value of W affects all three resonances of the antenna at different operation bands. According to the obtained results, it can be calculated that the antenna frequency response in all operation bands is very flexible to be tuned to lower or upper frequencies. In addition, its impedance matching can be also affected by changing the parameter values [57,58]. 

**Figure 5:**
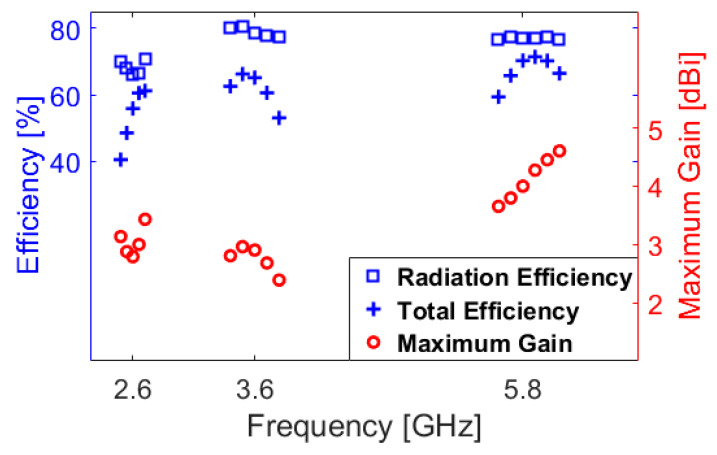
Radiation characteristics of the PIFA element versus its operation bands.

The fundamental radiation characteristics of the modified PIFA design including the radiation efficiency (R.E.), total efficiency (T.E.) and maximum gain (M.G.) are studied in Figure 5. In theory, the radiation and total efficiencies are related according to

*e* = *e_r_e_cd_* (1)

The antenna gain can be calculated using the radiation efficiency and the directivity as follows:

(2) $$G_{0}\left(dB\right)=10\log\left(e_{cd}D_{0}\right)$$

where *e*_0_ is the total efficiency, *e_r_* is the reflection (mismatch) efficiency = (1−|*Γ*|2), *e_cd_* is the radiation efficiency and *D*_0_ is the antenna directivity [59]. As can be observed from Figure 5, the antenna provides better than 40% and 65% radiation and total efficiencies over the three operation bands. In addition, the maximum gain of the design varies from 2.5 to 4.5 dBi.

## 4. Characteristics of the Handset Antenna Array

**Figure 6:**
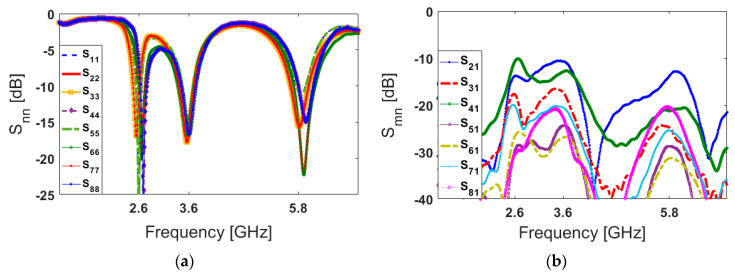
(**a**) S_nn_ and (**b**) S_mn_ properties of the proposed 5G handset antenna.

Figure 6 shows the S parameters of the designed handset antenna. As illustrated, the antenna exhibits good S parameters at three operation bands with acceptable mutual coupling at less than −10 dB [5,60]. According to the obtained results in Figure 6, slight variation can be observed in the S_nn_ results of the antenna elements, especially in the lower band (2.6 GHz). This variation is mainly due to different placements, feeding points, neighboring, and also unsymmetrical configurations of the employed elements in the main design (smartphone PCB), as shown in Figure 1. Besides this, the smartphone PCB is in a rectangular shape (with a size of 150 × 75 mm^2^) which could cause some discrepancies on the frequency responses and couplings, mainly between even and odd port numbers (antennas 1 and 2, for example). However, due to the flexible frequency behavior of the antenna elements (explained in Figure 4), by modifying the design parameters of the antenna element, the frequency response can be easily adjusted to the desired frequency bands [61].

**Figure 7:**
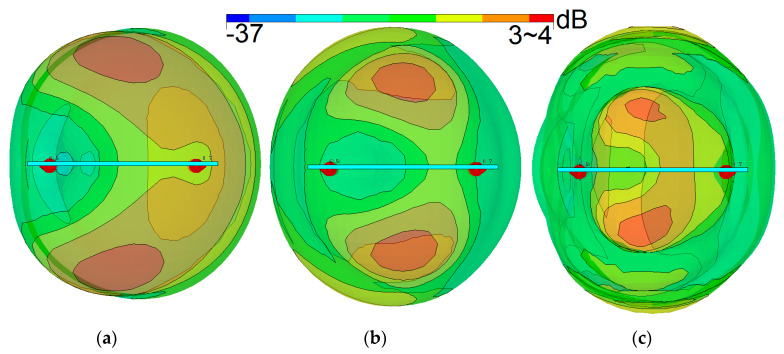
Side views of the antenna radiation patterns at (**a**) first (2.6 GHz), (**b**) second (3.6 GHz), and (**c**) third (5.8 GHz) resonances.

**Figure 8:**
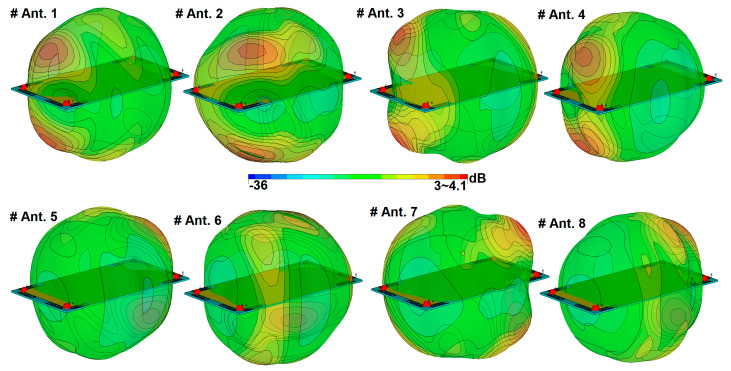
Radiation patterns of the PIFAs at the middle frequency (3.6 GHz).

The side view of the design radiation patterns for a single-element radiator at different operation frequencies is illustrated in Figure 7. Clearly, the antenna radiation elements exhibit high symmetric radiation patterns covering the different sides of the handset mainboard and increasing the radiation coverage [62,63,64,65]. The 3D radiation patterns for the eight PIFAs of the main design are displayed in Figure 8. As illustrated, the gain level of the design varies from 3 to more than 4 dBi. Besides, due to the placements of the PIFA element, four horizontally and vertically polarized radiation patterns are achieved to improve the MIMO performance of the design [66,67].

The envelope correlation coefficient (ECC) and total active reflection coefficient (TARC) characteristics are substantial in MIMO/diversity antenna systems [68,69]. These parameters can be extracted from the S-parameter results using the below formulas, respectively:

(3) $$ECC=\frac{{\left|S_{mm}^{*}S_{nm}+S_{mn}^{*}S_{nn}\right|}^{2}}{\left(1-{\left|S_{mm}\right|}^{2}-{\left|S_{mn}\right|}^{2}\right){\left(1-{\left|S_{nm}\right|}^{2\;}-{\left|S_{nn}\right|}^{2}\right)}^{*}}$$

(4) $$TARC=-\sqrt{\frac{{\left(S_{mm}+S_{mn}\right)}^{2}+{\left(S_{nm}+S_{nn}\right)}^{2}}{2}}\;$$

**Figure 9:**
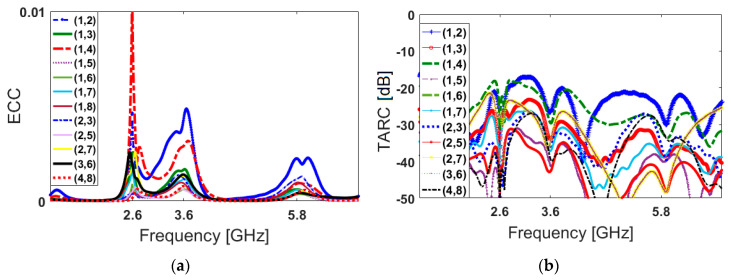
Calculated (**a**) ECC and (**b**) TARC results of the proposed design.

The ECC and TARC results of the presented multi-mode MIMO antenna design are calculated and plotted in Figure 9. As seen from Figure 9a, the calculated ECC results of PIFA pairs are very low over the entire multi-operation bands (less than 0.01). Additionally, it can be observed from Figure 9b that the TARC value of the diverse PIFA pairs is less than −20 dB at different frequencies.

## 5. Fabrication and Measurements

**Figure 10:**
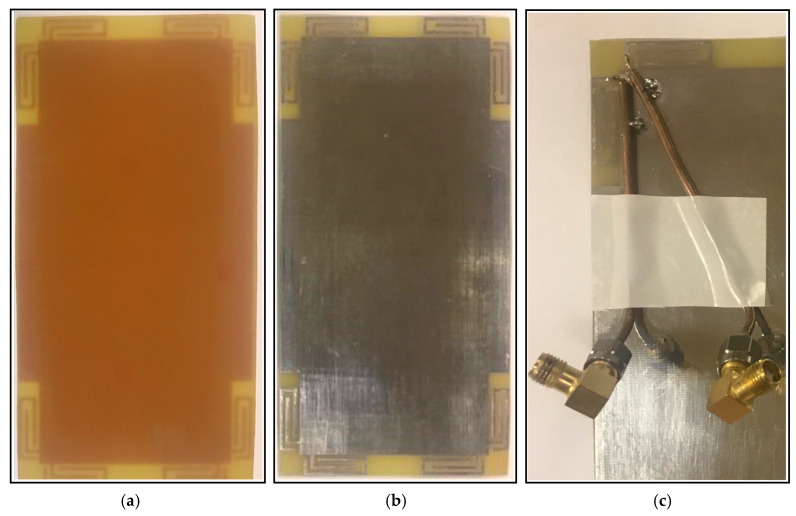
(**a**) Front and (**b**) back views, and (**c**) feeding mechanism of the fabricated sample.

A prototype sample of the proposed 5G handset antenna array was fabricated as illustrated in Figure 10a,b. Due to the similar placements and performances of the modified PIFA pairs, the properties of the handset antenna design for port 1 and 2 are measured and compared below. The feeding mechanism of the adjacent elements is shown in Figure 10c.

**Figure 11:**
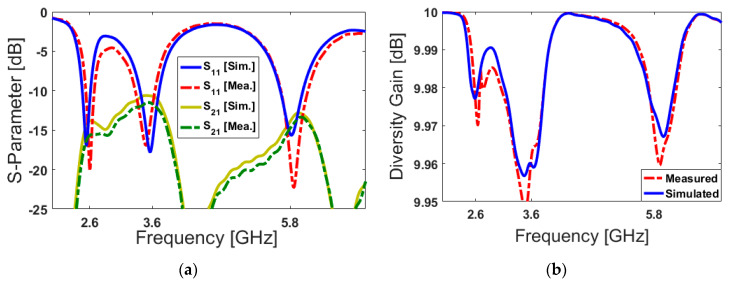
Measured and simulated (**a**) S-parameters and (**b**) diversity gain of the two adjacent PIFAs.

The measured and simulated results of the S-parameters are compared in Figure 11a. As seen, the S_11_/S_21_ measurements have good agreement with the simulated results in terms of covering the required multi-operation bands: a quite good impedance bandwidth (S_11_ < −10) is achieved to cover the operation bands of 2.45–2.65 GHz, 3.5–3.7 GHz and 5.6–6 GHz with resonances at 2.6, 3.6, and 5.8 GHz, respectively. In addition, as shown, the mutual couplings of the adjacent PIFAs are less than −15, −10, and −13 dB at the desired frequency bands. One of the vital parameters for the MIMO performance of an antenna array is diversity gain (DG), which can be calculated using the following formula:

(5) $$DG=10\sqrt{1-{\left(ECC\right)}^{2}}$$

**Figure 12:**
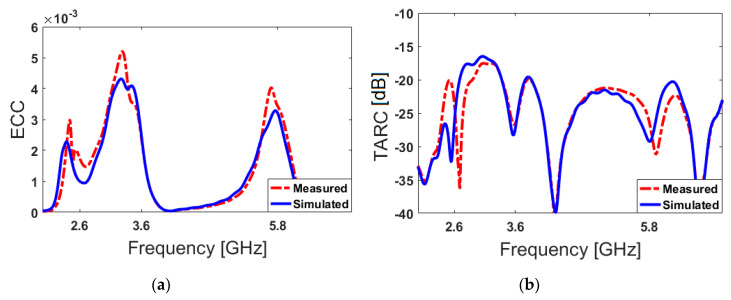
Calculated (**a**) ECC and (**b**) TARC results of the adjacent elements.

The DG characteristics of the antenna are illustrated in Figure 11b. The diversity gain function of the designed antenna over its operation band is more than 9.95 dB over the operating frequency bands [70]. In Figure 12a,b, we plot and compare the calculated TARC and ECC results of the PIFA pairs from simulated and measured results. As shown, the ECC function is very low (less than 0.05) over the different frequency bands of interest. Besides, the obtained TARC results are less than −18 dB.

**Figure 13:**
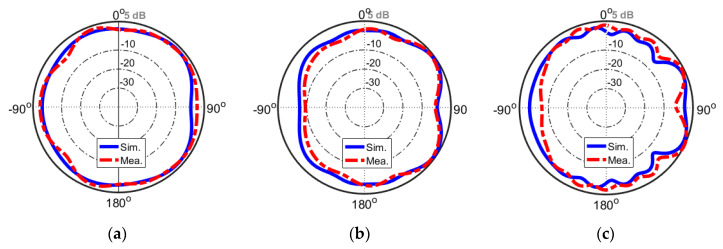
Two-dimensional radiation patterns at (**a**) first (2.6 GHz), (**b**) second (3.6 GHz), and (**c**) third (5.8 GHz) resonances.

Measured and simulated radiation patterns (H-plane) of the modified PIFA element at different frequencies are shown in Figure 13. It is worth noting that, during the measurement of the antenna radiation patterns, one port was kept excited while the other one was loaded with a 50 Ω load. As can be clearly seen, the sample handset antenna prototype provides good quasi-omnidirectional radiation patterns at different resonance frequencies with peak gains and acceptable agreement between simulations and measurements. It is found that when the antenna frequency increases, the gain level of the antenna is increased [71,72,73].

## 6. Comparison

Table 2 compares the characteristics of the proposed handset antenna design and some reported 5G smartphone MIMO antenna designs in the literature with the recent states of the art [16,17,18,19,20,21,22,23,24,25,26,27,28,29,30,31,32]. To the best of our knowledge, most of the reported sub-6 GHz 5G antennas are single-band operating, and only a few works have reported on the use of the dual-band or multi-band techniques for 5G smartphone applications. The main contribution of our work is that we propose a planar 8 × 8 MIMO diversity antenna array with a triple-band function covering the LTE 41, 42/43, and 47 bands. As clearly shown, compared with the recently introduced MIMO handset antenna systems with planar and uniplanar structures, our proposed handset antenna provides better characteristics in terms of impedance matching and bandwidth and low-profile radiators.

The proposed design achieves improvements not only around tri-band impedance bandwidth but also offers polarization diversity function at different edges of the mainboard. This is mainly due to orthogonal placements of the adjacent PIFA resonators at different corners of the PCB. In addition, unlike the reported handset antennas in the literature, the presented antenna is set on the single (back) layer of the platform which makes the design easy to integrate with the handset circuit. Furthermore, due to the small clearance of the proposed handset antenna, its characteristics in terms of data and talk modes are not changed significantly; that is, the proposed eight-element antenna array has the advantage of the comprehensive performance, meaning that it can be applied well in future 5G mobile terminals.

## 7. User Effects on the Characteristics of the Designed Antenna Array

**Figure 14:**
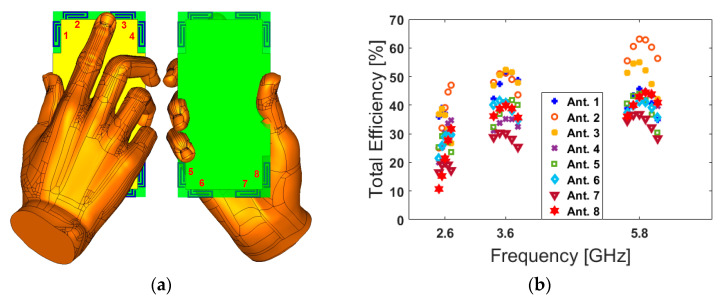

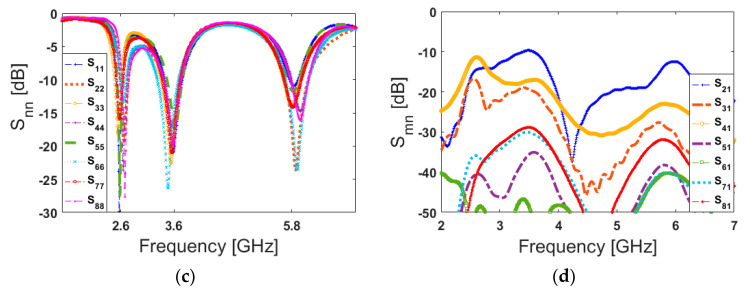
(**a**) Placement, (**b**) total efficiencies, (**c**) S_nn_, and (**d**) S_mn_ results for the right-hand scenario.

**Figure 15:**
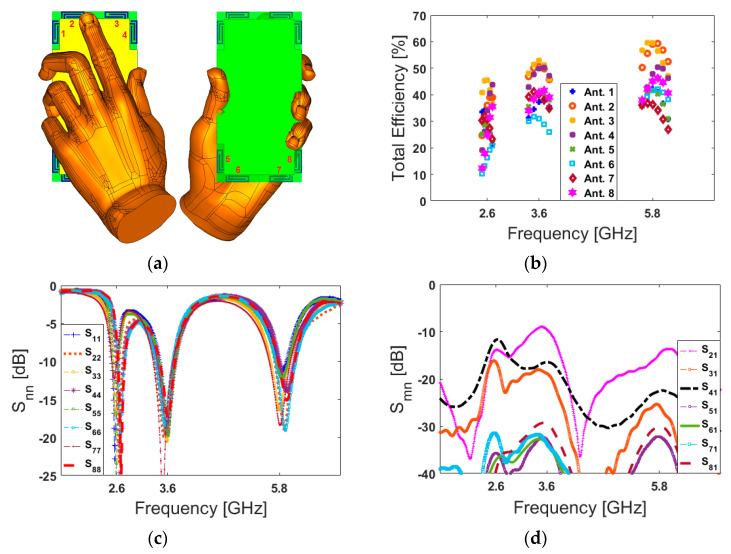
(**a**) Placement, (**b**) total efficiencies, (**c**) S_nn_, and (**d**) S_mn_ results for the left-hand scenario.

The health hazards of emitted electromagnetic (EM) radiation from mobile handsets has become a point of open deliberation as the use of mobile handsets is increasing exponentially [74]. For mobile handsets, the investigation of the user-effect on the characteristics of the antenna is indispensable [75]. Below, different usage postures in the data-mode of the user-hand for right and left hands are considered and studied in Figure 14 and Figure 15, respectively. The employed user-hand phantom in the simulation has a relative permittivity of ε = 24 and conductivity of σ = 2 s/m [76]. According to the obtained results, the proposed design exhibits similar radiation behavior for different hand scenarios. This is mainly due to the symmetrical schematic of the designed MIMO antenna. It is shown that the handset antenna and its modified PIFA elements exhibit good efficiencies. Besides, when the antenna frequency increases, the efficiency of the antenna is improved. The reflection coefficients (S_nn_) of the proposed array are not affected drastically by the hand, except for some small frequency fluctuations. However, the antenna efficiencies of the proposed array are affected significantly owing to the absorption effect of the user’s hand. This is because some EM energy has been absorbed by the hand [77]. Compared with the antenna performance in free space, the maximum reductions of the total efficiencies are observed for the PIFA elements that have been partially covered by the hand [78]. Additionally, as evident from the results, the antenna provides good S-parameters including S_nn_ and S_mn_ in both right and left-hand scenarios.

**Figure 16:**
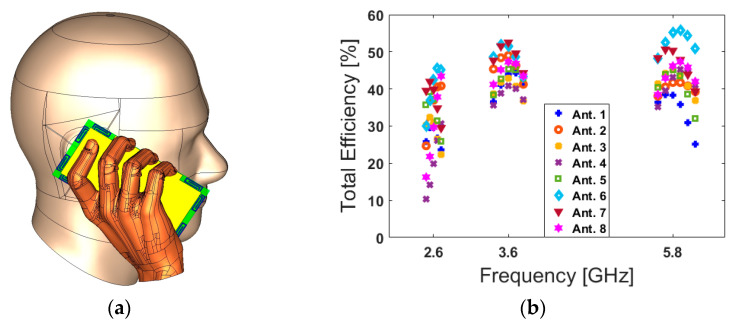

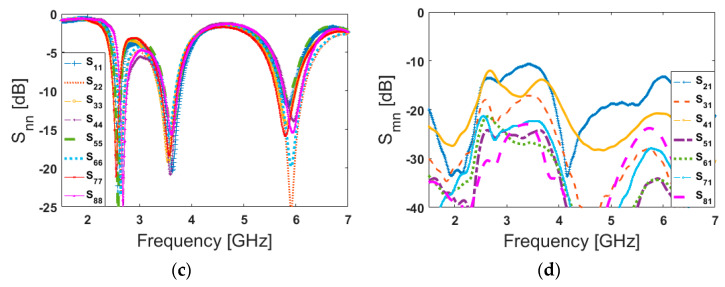
(**a**) Placement, (**b**) total efficiencies, (**c**) S_nn_, and (**d**) S_mn_ results for the talk-mode scenario.

Apart from the data-mode, the antenna performance in talk-mode should be also studied. Figure 16 shows the total efficiency, S_nn_ (S_11_–S_88_), and S_mn_ (S_21_–S_81_) characteristics of the designed handset antenna in the presence of the user-hand/user-head in talk-mode.

Figure 16a shows the placement of the designed antenna array in talk-mode; the array’s total efficiencies are represented in Figure 16b. It is shown that the antenna elements exhibit relatively good efficiencies at different resonance frequencies. Additionally, the antenna S-parameters are depicted in Figure 16d. As can be seen, the PIFAs are operating at the target frequencies with good S_nn_ and less than −10 dB S_mn_.

**Figure 17:**
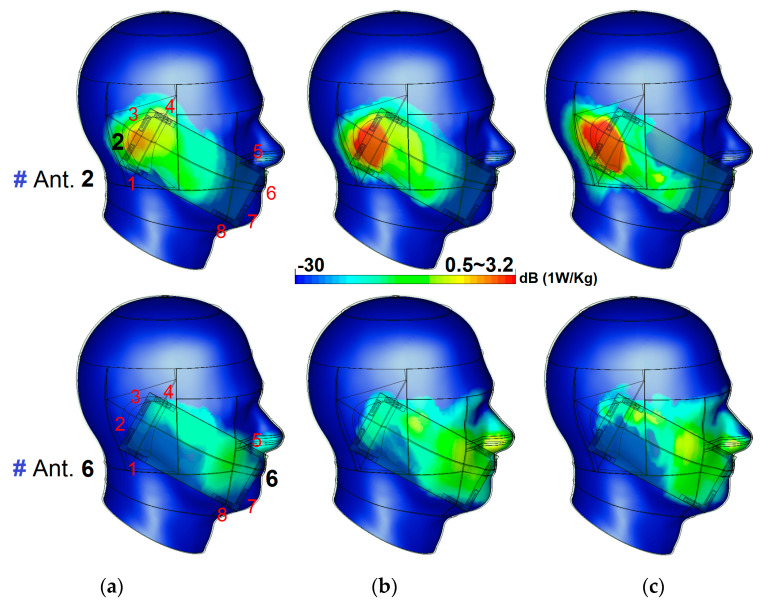
SAR for antennas 1 and 5 at (**a**) first (2.6 GHz), (**b**) second (3.6 GHz), and (**c**) third (5.8 GHz) resonances.

The EM energy absorbed by human body tissues can be evaluated by the specific absorption rate (SAR) [79,80]. SAR is a measure of how much power is being absorbed per unit mass. The SAR features of the MIMO design at three different operation frequencies are investigated. According to the obtained results from different investigations of the antenna elements, it is found that antenna 2 causes the maximum SAR value while the minimum SAR value is observed from antenna 6. The SAR results of antenna 2 and antenna 6 at 2.6, 3.6, and 5.8 GHz are depicted in Figure 17. According to the results, the distance between the PIFA elements and the head phantom is most important in terms of the value of the SAR function.

**Figure 18:**
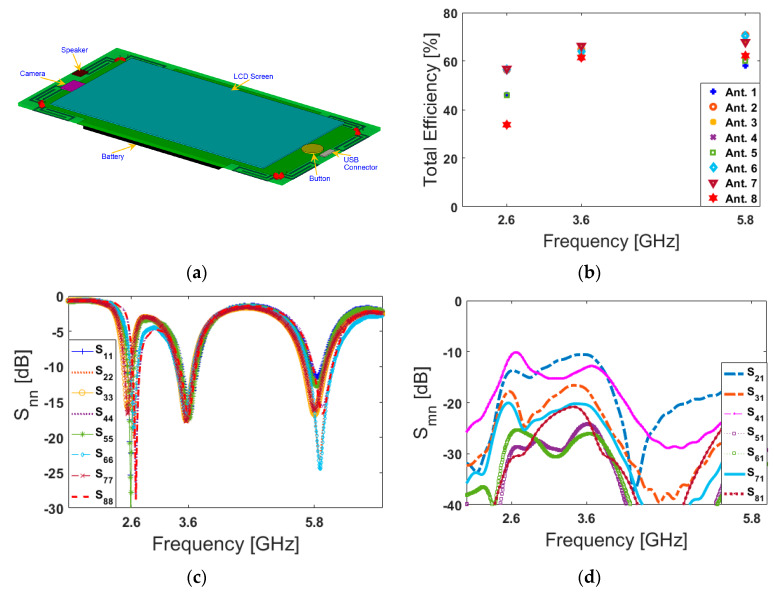
(**a**) Placement, (**b**) total efficiencies, (**c**) S_nn_, and (**d**) S_mn_ results in the presence of the smartphone components.

Below, the fundamental characteristics of the MIMO antenna including the total efficiency, S_nn_ and S_mn_ are also studied in the presence of smartphone components including the battery, speaker, camera, USB connector, and LCD screen. Table 3 lists the characteristics of the modeled components [81]. It is clearly seen from Figure 18 that the design provides consistent characteristics at the desired operating frequencies.

## 8. Integration of a Compact MM-Wave Phased Array

**Figure 19:**
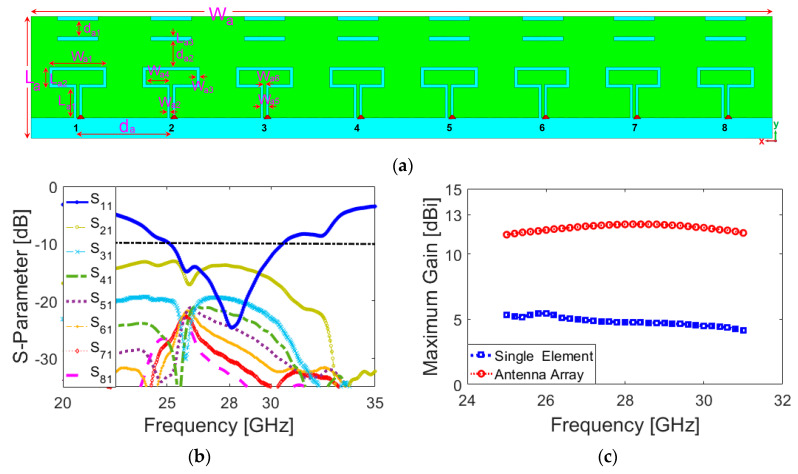
(**a**) Design details and configuration of the array, (**b**) its S-parameters, and (**c**) gain comparison of the array and the antenna element.

In this section, a new and miniaturized MM-Wave phased array 5G antenna with broad bandwidth is proposed to be incorporated in a shared board. The design details of the integrated phased array are illustrated in Figure 19a. It has a very compact size, with an overall size of W_a_ × L_a_ = 18 × 5 mm^2^, and it can be implemented in the same FR-4 laminate PCB with a thinner thickness of 0.8 mm. As shown, its configuration is composed of eight loop dipole resonators with pairs of directors arranged in a linear form. A discrete feeding port is applied separately for each antenna. The parameter values (in mm) are as follows: W_a1_ = 2.8, W_a2_ = 0.125, W_a3_ = 0.15, W_a4_ = 1.8, W_a5_ = 0.4, W_a6_ = 0.15, L_a1_ = 1, L_a2_ = 1.5, L_a3_ = 0.2, d_a_ = 0.125, d_a1_ = 0.8, d_a2_ = 1.3. The phased array is designed to work at 28 GHz—one of the promising 5G candidate bands at higher frequencies [82,83]. However, due to the broad bandwidth characteristic, the proposed phased array is also capable of covering 26 and 30 GHz 5G bands [84,85,86].

**Figure 20:**
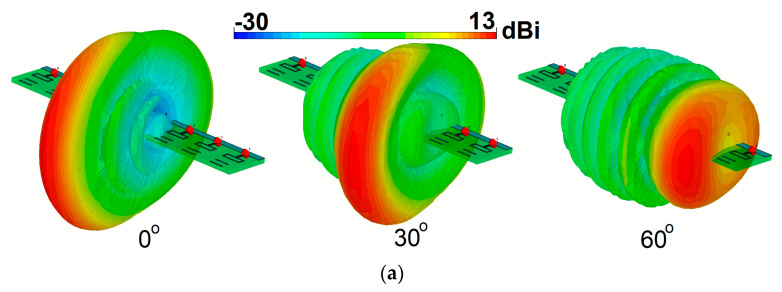

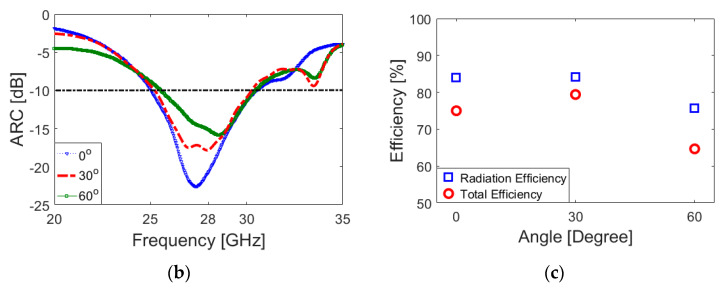
(**a**) Radiation beams, (**b**) active reflection coefficient, and (**c**) efficiencies at different scanning angles.

Figure 19b plots the S-parameters (S_11_–S_41_) of the phased array. As seen, the designed phased array provides a broad impedance bandwidth of 25–31 GHz with a central frequency of 28 GHz. Besides, less than a −15 dB mutual coupling characteristic is obtained for the antenna elements. Figure 19c compares the maximum gains of the antenna element and the phased over the operation band. It is shown that the antenna element provides 4~5 dBi gain values over the operation frequency, whereas more than 12 dBi maximum gain is achieved for the designed array. The 3D beam steering functions of the array at 28 GHz for different angles are plotted in Figure 20a. The shapes and directions of the phased array beams are determined by the relative phase amplitudes applied to each antenna element with discrete-feeding ports, as shown below [87]:

(6) $$\psi =2\pi \left(\frac{d}{\lambda }\right)sin\theta$$

As shown, the designed phased array provides a good beam-steering function with end-fire radiation beams. As illustrated, the designed array exhibits high gain radiation beams with low sidelobes.

**Figure 21:**
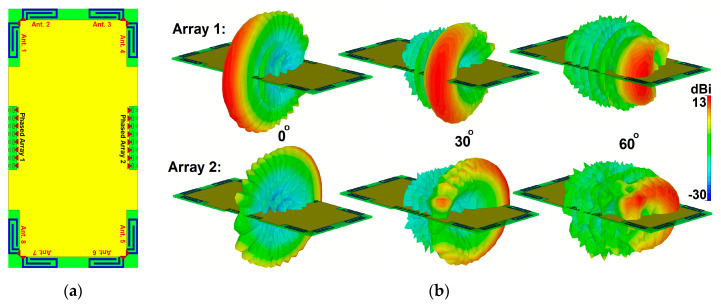
(**a**) Different placements of the proposed phased array into the smartphone board and (**b**) its beam steering function at different angles.

The active reflection coefficients (ARCs) of the design for different scanning angles are illustrated in Figure 20b. As the radiation beam of the antenna is scanned, the amount of coupling between the radiation elements changes, meaning that the active reflection coefficient curve moves slightly [88]. However, as shown, the proposed phased array antenna exhibits sufficient performance and supports the target frequency bandwidth for different scanning angles. The radiation efficiency of the proposed array antenna is more than 75% in the operating bandwidth of all switching modes. Figure 20c plots the radiation and total efficiency levels of the array at scanning angles of 0, 30, and 60. It is shown that the proposed phased array offers quite good efficiency results. Figure 21 shows the possible placements and radiation beam-steering of the proposed phased array in the configuration of the smartphone board. It can be observed that the array can be easily integrated into a small area of the PCB and could provide full radiation coverage supporting different sides of the handset [89].

## 9. Conclusions

The design and characteristics of a new MIMO smartphone antenna with a multi-mode operation is successfully investigated in this paper. The designed handset antenna contains eight modified PIFA elements deployed at four corners of the mainboard. The proposed design operates at 2.6, 3.6, and 5.8 GHz for sub-6 GHz 5G mobile terminals. It offers good characteristics in terms of bandwidth, isolation, and radiation patterns. In addition, quite good characteristics are observed in the presence of the user. Due to the tri-band and polarization diversity, the antenna can be considered for multi-mode future handset applications. Furthermore, due to the available space on the smartphone antenna system, a compact 28 GHz phased array is proposed to be integrated onto the 5G smartphone.

## Figures and Tables

**Table 1:** The dimension values of the presented multi-mode array.

Parameter	W_X_	L_X_	W_X1_	L_X1_	W_X2_	W	L	W_f_
**Value (mm)**	150	75	83	18	5	20.5	6.5	1
**Parameter**	L_f_	W_1_	L_1_	W_2_	L_2_	W_3_	L_3_	L_4_
**Value (mm)**	1	1	15.3	3.5	14.3	16	5.5	4.5

**Table 2:** Comparison of the design characteristics with the referenced handset antennas. MIMO: multiple-input–multiple-output.

Reference	Antenna Type	Elements	Bandwidth (GHz)	Efficiency (%)	Overall Size (mm^2^)	Isolation (dB)	ECC
**Single-Band MIMO Handset Antennas**							
[16]	Coupled-Fed	8	2.55–2.68	48–63	136 × 68	12	<0.15
[17]	Petal Slot	8	2.55–2.66	-	150 × 75	10	<0.1
[18]	Loop	8	2.55–2.6	48–63	136 × 68	11	<0.15
[19]	PIFA Slot	8	3.4–3.6	62–78	140 × 70	10	<0.20
[20]	Slot-Ring	8	3.4–3.8	55–70	150 × 75	15	<0.05
[21]	Patch-Slot	8	3.55–3.65	52–76	150 × 75	11	-
[22]	Loop Element	8	3.3–3.6	40	120 × 70	15	<0.02
[23]	CPW-Fed Slot	8	3.4–4.4	65	150 × 75	16	<0.01
[24]	Monopole	8	4.55–4.75	50–70	136 × 68	10	-
**Dual-Band MIMO Handset Antennas**							
[25]	Coupled-Fed Slot	10	3.4–3.8	41–84	150 × 80	12	<0.15
			5.15–5.92	47–79			
[26]	Dual Mode Monopole	2	3.4–3.6,	50–61,	150 × 76.6	10.5	<0.2
			5.725–5.875	67–80			
[27]	Cuboid Monopole	6	2.6–2.7	60–70	140 × 05	10	<0.05
			5.1–5.9	70–80			
[28]	Monopole	8	3.2–3.9	60–80	150 × 75	12	-
			5–5.5	70–85			
[29]	Folded Monopole	8	2.45–2.6	40–60	124 × 74	15	<0.2
			3.45–3.65	50–80			
**Tri-Band MIMO Handset Antennas**							
[30]	Coupled-Fed Arm	8	3.3–3.8,	55–72,	130 × 70	10	<0.1
			4.8–5,	50–65,			
			5.1–5.9	43–73			
[31]	Yet-Decoupled Antennas	2	2.40–2.48	44–48,	150 × 75	15	<0.14
			5.15–5.35,	74–80,			
			5.72–5.92	75			
[32]	F-Shaped Monopole	4	3.3–4.2,	60–80	150 × 75	12	<0.1
			4.4–5,				
			5.15–5.85				
**Proposed**	**Modified Diversity PIFA**	**8**	**2.45–2.65,**	**40–65,**	**150 × 75**	**11**	**<0.01**
			**3.4–3.75,**	**50–70,**			
			**5.6–6**	**60–80**			

**Table 3:** Characteristics of the smartphone components.

Component	Material	Permittivity
Screen	LCD film	4.8
Battery. Camera, Speaker	perfect electric conductor (PEC)	-
USB Connector	Brass (PEC)	
Button	Rubber	3.5
printed circuit board (PCB)	FR-4	4.4
